# Virtual Screening Models for Prediction of HIV-1 RT Associated RNase H Inhibition

**DOI:** 10.1371/journal.pone.0073478

**Published:** 2013-09-16

**Authors:** Vasanthanathan Poongavanam, Jacob Kongsted

**Affiliations:** Department of Physics, Chemistry and Pharmacy, University of Southern Denmark, Odense M, Denmark; University of Pittsburgh, United States of America

## Abstract

The increasing resistance to current therapeutic agents for HIV drug regiment remains a major problem for effective acquired immune deficiency syndrome (AIDS) therapy. Many potential inhibitors have today been developed which inhibits key cellular pathways in the HIV cycle. Inhibition of HIV-1 reverse transcriptase associated ribonuclease H (RNase H) function provides a novel target for anti-HIV chemotherapy. Here we report on the applicability of conceptually different *in silico* approaches as virtual screening (VS) tools in order to efficiently identify RNase H inhibitors from large chemical databases. The methods used here include machine-learning algorithms (e.g. support vector machine, random forest and kappa nearest neighbor), shape similarity (rapid overlay of chemical structures), pharmacophore, molecular interaction fields-based fingerprints for ligands and protein (FLAP) and flexible ligand docking methods. The results show that receptor-based flexible docking experiments provides good enrichment (80–90%) compared to ligand-based approaches such as FLAP (74%), shape similarity (75%) and random forest (72%). Thus, this study suggests that flexible docking experiments is the model of choice in terms of best retrieval of active from inactive compounds and efficiency and efficacy schemes. Moreover, shape similarity, machine learning and FLAP models could also be used for further validation or filtration in virtual screening processes. The best models could potentially be use for identifying structurally diverse and selective RNase H inhibitors from large chemical databases. In addition, pharmacophore models suggest that the inter-distance between hydrogen bond acceptors play a key role in inhibition of the RNase H domain through metal chelation.

## Introduction

According to a recent report from the UNSAIDS, it is estimated that more than 34 million people are living with a HIV-1 type infection worldwide and 2.5 million new HIV infections occur every year. Currently, 14.8 million people are eligible for HIV treatment, however only 8 million people are under treatment due to various reasons which includes economical issues [1]. Although AIDS related mortality has been reduced by 24% (1.7 million in 2011) compared to 2005 data (2.3 million), development of improved anti-HIV regiments is still required. To control HIV progression, several viable chemo-targets have been identified [2], [3] in the HIV replication cycle, such as fusion or entry of HIV with the host CD4 receptor, reverse transcription of viral RNA into viral DNA (by reverse transcriptase), integration of viral DNA with host DNA (by integrase), and maturation of new viral protein (by protease). Although significant improvements have been made in HIV therapy within the last decades, there is still a strong demand for improving AIDS therapy due to an increasing drug resistance [4], [5]. Reverse transcription of genomic single strand RNA into double strand DNA (dsDNA) by viral reverse transcriptase (RT) is a key process in replication of HIV and dsDNA is subsequently integrated into the genome of the host cell. RT has two catalytic domains in order to carry out the reverse transcription process. Very briefly (1) the DNA polymerase domain uses cellular RNA primer (specifically tRNA^lys3^) to synthesize single strand viral (−) DNA (RNA dependent DNA synthesis), subsequently, the synthesized HIV (−) DNA is hybridized with a viral RNA template to form a RNA:DNA hybrid duplex, (2) RNase H domain removes the RNA strand from the hybrid and facilitate the first strand transfer which leads to formation of purine rich sequence of HIV RNA (also called “polypurine tract” (PPT). Here, PPT serves as a primer for the synthesis of viral (+) DNA strand and subsequently the RNase H removes the PPT portion after priming of (+) DNA synthesis. The majority of the currently marked antiviral drugs that have been approved by FDA (The Food and Drug Administration) for the treatment of HIV infection are RT inhibitors which particularly inhibits at the polymerase domain [6]. Due to the high rate of viral mutation and resistance to current drug regiments [7], considerable attention has in recent years been paid to less explored target sites within the HIV replication process [8]–[10], one such target is the inhibition of RT associated RNase activity [11].

**Figure 1:**
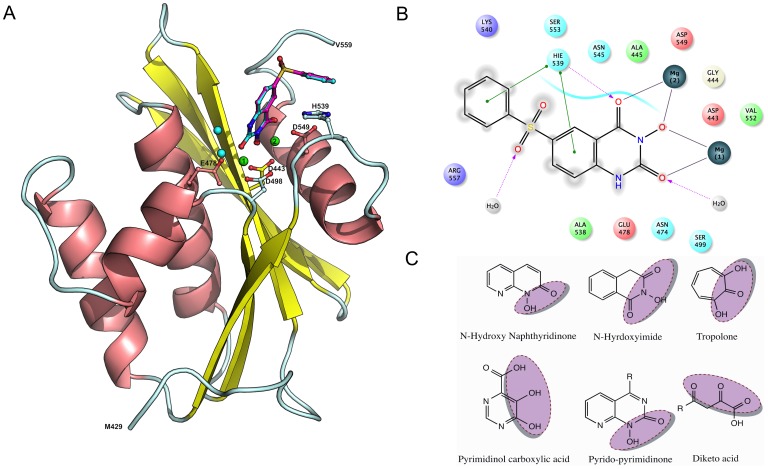
HIV-1 RT associated RNase H model and ligands structures. Panel A: The model of HIV-RT associated RNase H domain is shown with a bound ligand (cyan, ball and stick model). The catalytically important residues, magnesium ions (green sphere) and bound water (cyan sphere) have been highlighted. The docking pose of the bound ligand is shown in pink (ball and stick model), Panel B: 2D representation of ligand-protein interaction. Panel C: Representative molecules belong to the class of site directed RNase H inhibition.

RNase H is one of the two domains of the p66 (66 kDa) subunit of reverse transcriptase. From mutation and x-ray crystallographic studies [12]–[15] the structure of the RNase H domain has been well characterized. It is composed of five standard mixed sheets, which are surrounded by four helix, and eight loops in the center of the domain. The active site of RNase H consists of four highly conserved amino acids Asp-443 (D443), Glu-478 (E478), Asp-498 (D498) and Asp-549 (D549) (Figure 1A/B) and two catalytically active magnesium ions (Mg^2+^). Moreover, it has been shown that mutation of any of these residues abolishes the enzyme activity. This is because these residues provide a favorable environment for stabilizing metals (Mg^2+^), which is essential for a proper binding and positioning of the RNA:DNA duplex during the digestion process [16]. Over the past years a large number of RNase H inhibitors have been designed, synthesized and some entered into the clinical trial, but none of these have yet reached the market. Furthermore, these inhibitors all show a low level of specificity towards RNase H, meaning that many of these compounds also inhibit polymerase [17]–[19]. Two mechanisms of RNase H inhibition have been proposed; (i) compounds that blocks metal binding at the active site, (also called “active site directed RNase Inhibition”) and (ii) compounds that binds at the allosteric site of RNase H [20]. Both classes of compounds show high level of ligand diversity in general, however, the active site directed RNase H inhibitors are unique in the sense that the majority of inhibitors are rich on hydrogen bond acceptor sites, which is believed to form chelation with the magnesium ions [14], [15], [21]–[23]. Representative examples of molecules belonging to this class of site directed RNase H inhibition is shown in Figure 1C.

With an increasing number of high resolution 3D structures of HIV-1 RT associated RNase H domain and deposit of known inhibitor data in public databases, ligand and structure-based modeling tools may readily be used for designing selective inhibitors. Because of the rapid advancement in high throughput screening (HTS) approaches, the availability of screened compounds is increasing exponentially; for example, Parniak et al. screened nearly 100000 NCI chemical compounds for the HIV-1 RNase H inhibitor and deposited these at the PubChem database (AID: 372) [24]. This enormous information can subsequently be analyzed and trained for new lead identification through cheminformatics and bioinformatics approaches, e.g. a fingerprint based-decision tree method has been applied to classify inhibitors of the RNase H with success rate of approximately 99% for 10-fold cross validation (sensitivity = 57%, specificity = 99%) [25]. Very recently, ligand based virtual screening was successfully performed using shape-based similarity searching methods [26] for identification of duel inhibitors of HIV-1 RT. Subsequently the models were applied to screen the NCI chemical database and finally found 4 out of 34 molecules to shown inhibition at micro molar concentrations (2–22 µM). Thus computer-aided approaches such as molecular docking, 3D-QSAR, pharmacophore modeling, machine learning methods and shape-similarity based virtual screening has emerged as effective methods for identification novel compounds [27]–[29]. It has previously been observed from validation of virtual screening experiments that docking methods performed best in identifying high affinity CYP1A2 inhibitors compared to machine learning based screenings (correctly predicted 21 out of 41 tested). However, the models developed with random forest were found to be very efficient since it required only 39 two-dimensional physicochemical descriptors (or properties) for new prediction [27].

The aim of the present study is to develop virtual screening models for HIV-1 RT associated RNase H inhibition through conceptually different computational methodologies, for instance, using ligand and structure information. To this end, five screening methods have been evaluated and it is suggested which of these methods is best suite for efficient identification. The methods applied comprises (1) pharmacophore modeling, (2) ligand flexible docking, (3) grid based pharmacophoric -fingerprint for ligand and proteins (FLAP), (4) machine learning algorithms, (such as random forest, support vector machine, kappa nearest neighbor) (5) rapid overlay of chemical structures (ROCS). This is, to our knowledge, the first time that such conceptually different methodologies have been assessed with respect to efficacy and efficiency in virtual screening setup for identification of RNase H inhibitor of HIV-1 reverse transcriptase.

## Materials and Methods

### Ligand preparation

**Figure 2:**
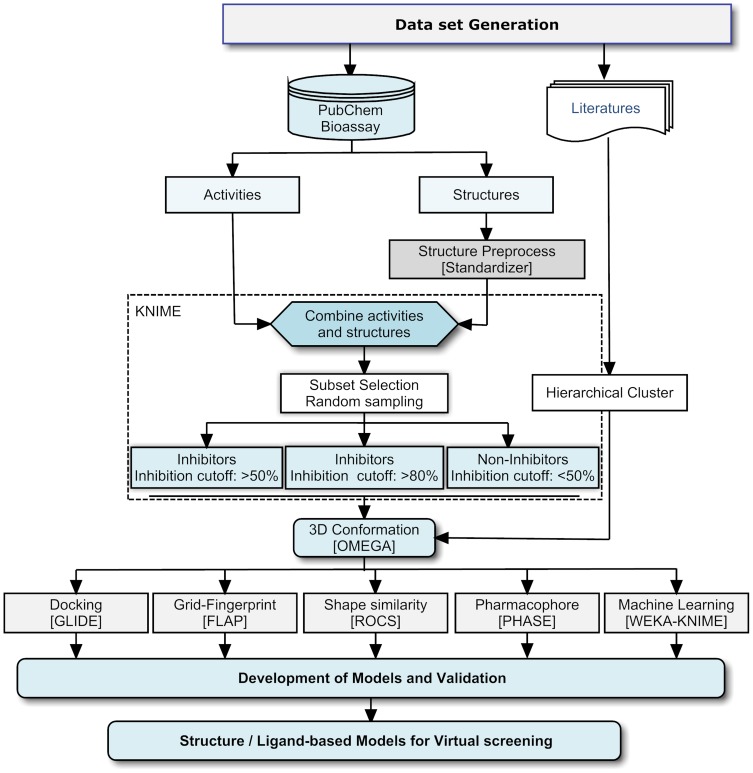
The overall workflow of the present study.

A set of 135 HIV-1 RT associated RNase H inhibitors was collected from the literature [14], [15], [22], [29]–[40]. This dataset consists of different scaffolds including highly active pharmacophores as represented in Figure 1C. In addition to these literature compounds, structure and biological activities (reported in binary format, i.e active as 1 and inactive as 0) of 99766 compounds were collected from the PubChem Bioassay database (AID: 372) [24]. In brief, the inhibition of HIV-1 RT associated RNase H catalyzing the RNA:DNA duplex (substrate) to fluorescein labeled-RNA strand was measured. Fluorescein tagged substrates show very low background fluorescence and upon hydrolysis the fluorescence increases up to 50 folds. Compound inhibition is thereby proportional to the fluorescence intensity [41]. Of the 99766 compounds retrieved from PubChem, 1863 compounds were removed, due to lack of either activity data or structures and in come cases the structures existed as duplicate entries. The remaining structures were imported into the Standardizer tool [42] (version 5.12.2) in order to refine the structures by normalization of chemo-types (e.g. tautomerization, aromatization and remove steric clashes). The remaining 97903 compounds, which includes 760 active and 97143 inactive, were processed for further steps. The overall workflow of the present study is shown in Figure 2.

### 3D conformation generation

Both set of compounds (literature and PubChem) were converted into 3D structures using OMEGA [43] (a conformation generating tool). As default OMEGA reports multi-conformer for all compounds using the MMFF95S force field, however, in the present study only a single conformer i.e. the one possessing the lowest energy was used.

### Subset Selection

Bioactivities and structures were imported into KNIME (version 2.6.3) [44], an open source data integration platform, which provides a variety of data mining/chemistry applications and data analysis nodes. In this study we used KNIME for subset selection purposes as well as for machine learning model developments. In order to reduce the computational time, we selected a small subset of inactive (n = 971 compounds, “Subset_inactive”) of the PubChem dataset, which represents the whole inactive in the dataset. The selection was made randomly as implemented in KNIME and activity threshold for the inactive compound was 0–50 (% of inhibition). Of the 760 active compounds in the PubChem dataset, 20 highly active and structurally diverse compounds were randomly chosen using KNIME (activity threshold >80% of inhibition) and stored for enrichment study as known binder (n = 20, “Subset_actives”). Structures and bioactivities of subsets used in this study are provided in the supplementary material.

### Template selection

Canvas (version 1.5) chem.-informatics tool [45] was used to perform a hierarchical clustering based on the dendritic fingerprints and Tanimoto similarity (TS) as method of metric (TS_AB_ = c/a+b−c, a = bits set to 1 in molecule A, b = bits set to 1 in molecule B, c = number of 1 bits common to molecule A and B). From the 135 compounds in the literature dataset, 22 individual clusters were obtained with merging distance cutoff 0.80. This was reduced to 15 clusters (merging distance cutoff 0.93) and two highly active compounds (in terms of pIC50) from each cluster were selected as template (n = 30, “subset_active_lit”) for pharmacophore search and enrichment study. A representative compound for each cluster as well as the full list of compounds used as known actives for validation is provided in the supplementary material [Figures S1, S2]. Special care was taken to verify that the x-ray bound ligands were also part of the subset selected as template.

### Machine learning methods and Feature selection methods

Various commonly used machine learning (ML) techniques such as support vector machine (SVM), kappa nearest neighbor (kNN) and random forest (RF) were applied. These methods also represent common approaches for classification. A detailed account of the theory behind these methods can be found elsewhere [46]. All the classification models were constructed using Weka [47], which provides a set of classification and regression methods, and attribute selection methods. In the present study, various automatic feature selection procedures were applied such as CfsSubsetEval (correlation-based feature subset selection evaluator) with BestFirst and Genetic search algorithms. All machine learning based classification and attribute selections used in this study were performed with Weka nodes as implemented in KNIME.

### Molecular descriptors and fingerprints

Ligand information for classification was obtained from molecule descriptors and fingerprints. A set of 494 two-dimensional descriptors and 269 fingerprints (including MACCS structural keys and Sub-structure fingerprints) were calculated using PaDEL [48], a descriptor computing software as implemented in KNIME. Descriptors used in this study includes physical properties, atom and bond counts, adjacency and distance matrix descriptors containing BCUT and GCUT descriptors, Kier & Hall connectivity, kappa shape indices, subdivided surface areas, and pharmacophoric features. Fingerprints represents small “sub-structures” consisting of one to ten non-hydrogen atoms and enumerates very simple features, but when used in combination they can prove to be very specific and useful in distinguishing the characteristics among the small molecules. Low variance descriptors (variance upper bound set to 0.0), and insignificant fingerprints (bits) were removed before the model optimization.

### Pharmacophore modeling

Pharmacophore modeling was carried out using the Phase (version 3.1) [49] module of the Schrödinger molecular modeling suits [50]. Phase uses LigPrep for the structure cleaning process, which includes generating possible tautomers, and ionization states (at pH 7), followed by generating conformers using the “ConfGen” macro-model search algorithm with thorough sampling. The OPLS2005 force field was chosen with a distance-dependent dielectric solvent model. Phase develops the pharmacophore hypothesis in three steps; (1) *creation of pharmacophoric sites* from active compounds using six common features: hydrogen bond donor (D), hydrogen bond acceptor (A), hydrophobic region (H), negatively charged region (N), positively charged region (P) and aromatic rings (R), (2) *perceiving common pharmacophore*, group similar pharmacophores according the inter-site distance (the distance between pairs of sites in the pharmacophore) using tree-based partitioning methods, and (3) finally, the surviving hypothesis from these partitioning procedures will be *scored and ranked*. The quality of the pharmacophore alignment was assessed using the RMSD (distance tolerance set to 1.2 Å) and the quality of the hypothesis assessed using survival score, sites, vector, and selectivity scores [see supplementary material for score description]. In the current setting, at least 4 common pharmacophore points must match with the selected active compounds and other settings were chosen as defaults.

### Structural similarity search

The rapid overlay of chemical structures (ROCS) software [51] from OpenEye was used for shape-based structural similarity search [52]. Shape similarity can be determined, in part by comparing the shape of a query molecule with a reference molecule. This program is particularly designed to screen a large database using a solid-body optimization process (uses only heavy atoms for superposition) that maximizes the molecular overlap (volume and atomic features). ROCS uses the ImplicitMillsDean force field that defines six pharmacophoric color fields, such as hydrogen bond donor, hydrogen bond acceptor, hydrophobic region, anions, cations, and rings. Once the overlap is optimized, the shape similarity is computed using TanimotoCombo (ranges 0–2, high score indicates the higher shape and color similarity). TanimotoCombo provides the scores that include both shape similarity fit and color similarity fit (chemical pattern). The ROC program reads SMILES, 2D, or 3D structures as input and generates possible low energy conformations using the OMEGA 3D structure generating module as implemented in ROC.

### Fingerprint for Ligands and Proteins (FLAP)

The software FLAP [53] was used to build and validate ligand based virtual screening models. FLAP uses fingerprints derived from GRID molecular interaction fields (MIFs) and GRID atom types are characterized as quadruplets of pharmacophoric features. The GRID approach is a well assessed concept for determining energetically favorable interaction sites in molecules with known structure using chemical probes e.g., H, O, N1, and DRY probes which describes the shape, hydrogen bond acceptor, hydrogen bond donor and hydrophobic interactions, respectively. FLAP creates a common reference framework in two steps: first, the MIFs of the molecules are calculated using the GRID force fields, and the resulting MIF's are condensed in complexity by extracting points (quadruplets or hotspot) representing the most favorable interactions. Second, each quadruplet of these points is used to generate different superpositions of the test molecules onto a template molecule. The quadruplets of each molecule are stored as a pharmacophoric fingerprints and used to evaluate their similarity. Superposition of quadruplets is assessed through Probe scores and Distance score, which represents the degree of overlap of the MIFs for each probe individually as well as for their combinations and overall difference of probe score between the ligand and template, respectively. In addition, FLAP calculates Global Sum score (Glob_Sum) and Global Product scores (Glob_Prod). The former score is produced by summing all the scores of the individual probes together and the later score is produced by multiplying all the scores of the individual probes together. All similarity measures ranges from 0.0 (bad) to 1.0 (good), except the Distance, where 0 is good. GRID probes H, N1, DRY and O were used for the FLAP modeling. The rational choice for these four specific probes is justified by the fact that they represent distinct ligand-protein interaction features. The distance (i.e. spatial resolution) between two GRID points was set to 0.75 Å.

### Automated docking and scoring: preparation of protein and ligands

A computational model of the HIV-1 RT associated RNase H domain was constructed from an X-ray crystal structure with resolution of 1.4 Å from the Protein Data Bank (PDB ID: 3QIO) [15]. In the deposited crystal structure of RNase H domain with N-hydroxy quinazolinediones (bound active site inhibitor), 12 residues were missing and the structure was determined with manganese (Mn^2+^) ions instead of the two catalytically active magnesium ions. Atomic coordinates for the missing residues were generated using the Swiss-Model [54]. Subsequently, the protein model was imported into the Maestro module available in the Schrödinger package and the protein was further optimized using the Protein Preparation Wizard [55]. This optimization includes adding hydrogen atoms, assigning correct bond orders and building di-sulfide bonds and replacing the Mn^2+^ ions with Mg^2+^ ions. The protonation states of all of the ionizable residues were predicted by PROPKA [56] provided in the Protein Preparation Wizard in the presence of the Mg^2+^ ions at the active site. An optimized structure model was energy minimized (only hydrogen atoms) using the OPLS2005 force field.

The receptor grid generation module of Glide [57] was used to define the active site for the docking experiments. As this protein model has a bound ligand (3-hydroxy-6-(phenylsulfonyl)quinazoline-2,4(1H,3H)-dione), the ligand was set as the centroid of the grid box (size of the active site is 20 Å from ligand position). Water molecules in the active site beyond 3 Å from the bound ligand were deleted.

### Ligand flexible docking protocol

Glide (version 5.8), a grid-based exhaustive search algorithm was used for all docking experiments [57]. Glide uses a series of hierarchical filters to find possible ligand pose in the active site, and the program has the option to treat the ligand fully flexible or rigid during the docking run. In addition, glide provides three docking precision modes, namely, XP (extra precision), SP (Standard precision) and HTS (High-throughput screening) modes. Each mode are used in slightly different context, e.g., the HTS mode is used to screen a relatively large database (uses more restricted conformational sampling), the SP mode uses a softer scoring function that adapt at identifying ligands that have a reasonable propensity to bind in the receptor, and the XP mode uses a complete minimization, and scoring (and additional terms used over SP, e.g. solvation) from large ensembles of docking poses (requires more CPU time), thus this mode is specially used for top-ranked compounds. Glide uses an in-build docking scoring function resulting in a Glidescore (SP and XP). In the current setting, all three docking modes were analyzed for the virtual screening. Together with the different ligand preparation settings (original and Epik process [58] from LigPrep) this resulted in a total of 6 docking runs (Table 1) for all sets of ligands in the dataset. Epik is an application that generates possible protonation states, tautomers and metal binding sites in the ligand.

**Table 1:** Summary of enrichment factor estimation from various docking scenarios.

Enrichment run	Models	Dataset	Number of compounds		Docking Scenarios	Enrichment factor			ROC
			Active	Inactive		1%	2%	5%	
Pubchem Actives	1	Original	20	971	HTS	20	11	8.5	0.82
	2				SP	29	15	8.9	0.91
	3				XP	24	15	8.0	0.72
	4	Epik	36	1526	HTS	21	12	6.0	0.90
	5				SP	20	12	6.0	0.90
	6				XP	15	9.9	4.0	0.77
Literature Active	7	Original	30	971	HTS	29	24	9.9	0.81
	8				SP	32	27	11.0	0.83
	9				XP	29	17	9.2	0.68
	10	Epik	59	1526	HTS	31	17	8.7	0.88
	11				SP	30	21	10.0	0.89
	12				XP	10	13	8.6	0.83
Combined Active datasets	13	Original	50	971	HTS	20	19	8.7	0.81
	14				SP	20	20	10.0	0.87
	15				XP	20	14	8.7	0.70
	16	Epik	95	1526	HTS	20	14	7.6	0.89
	17				SP	20	16	8.0	0.90
	18				XP	12	10	6.8	0.81

Note: ROC = Receiver Operator Characterics, XP: Extra precision, SP: Standard precision, HTS: High throughput screening.

### Validation of virtual screening models

The computational models of each experiment were validated according to their assessment parameters, e.g., the pharmacophore goodness was evaluated according to its survival score. The quality of the machine learning models was assessed using the terms, *Sensitivity* (the proposition actives being predicted as actives), *Specificity* (proposition of inactive being predicted as inactive), *G-mean* (a measure of balanced prediction of each of the two classes) and *Matthews's correlation coefficient (MCC)* (The quality of the overall binary classification model and models are considered to be predictive if MCC is higher than 0.4.).
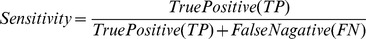
(1)

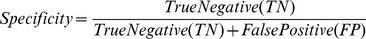
(2)

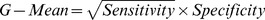
(3)

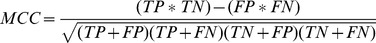
(4)In addition, 10-fold cross validation was also carried out as implemented in Weka from KNIME. The virtual screening effectiveness is evaluated in terms of the enrichment factor (EF) as a function of the percentage×that is covered of the complete ranked database, meaning that the proportion of active molecules found from a test database in which a small number of actives have been hidden in a large inactive compounds. Thus,
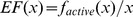
(5)where f_Active_ is the percentage of the actives found after assessing x% of the ranked database. Successful screening implies EF>>1. In addition to the EF, the enrichment is also assessed with a receiver operating characteristic (ROC) value and area under the curve (AUC). This denotes how good the model is in discriminating active compounds from inactive. ROC/AUC values range from 0.0 (poor) to 1.0 (ideal) and 0.5 represents a random prediction.

## Results and Discussion

As mentioned in the introduction the aim of the current study is to develop and validate virtual screening models that can efficiently be used to find highly selective and potent HIV-RT RNase H inhibitors. To achieve this goal, we used conceptually different *in silico* methods as detailed above. Below, we will present and discuss our virtual screening experiments for efficient identification of inhibitors for the targeted protein.

### Characterization of the dataset

**Figure 3:**
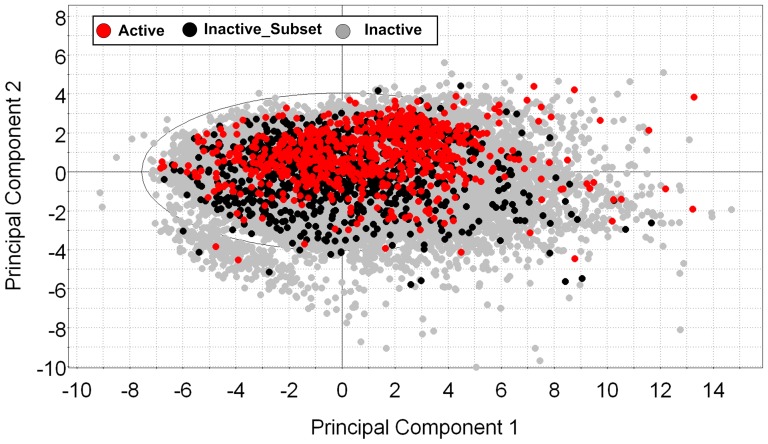
HIV-1 RNase H ligands chemical space. Scatterplot from principal component analysis (first two PCs). Inhibitors are shown in red, the subset of non-inhibitors is shown in black, and the remaining non-inhibitors are shown in gray.

The drug-likeness of the dataset was assessed by calculating the Lipinski's “rule-of-five” descriptors and count how many of these that fail each rule. The result shows that >97 of the compounds in the dataset did not violate any of the four properties (e.g., hydrogen bond donor, hydrogen bond acceptor, logP, molecular weight) and only 3% of the molecules in the dataset violates one or two rules. As mentioned in the method section developing models with large number of compounds (e.g., ∼97000) significantly reduce the computational efficiency and highly imbalanced class distribution, which leads to over prediction of major class, e.g., active (minor class) predicted as inactive (major class). Thus we decided to select a subset of the compounds which represents the rest of the active and inactive compounds in the whole dataset. A subset consisting of 971 inactive compounds was selected using random sampling from KNIME, and projected to the rest of the inactive compounds in the dataset to check the chemical space of this subset and the rest of the compounds in the dataset using a principal component analysis (PCA). The PCA was computed using the SIMCA-P program (Version 10.0. Umetrics, Umeå, Sweden). It was observed from the scatter plot (Figure 3) of the first two PCs (R^2^ = 0.69, Q^2^ = 0.59) that there is a small cluster located in the lower left part of the scatter plot and inspection of these compounds reveals that there is no structural similarity within this cluster. In addition, the scatter plot suggests that the subset (n = 971) clearly reflects the whole dataset. This indicates that this subset could be used for validation (as inactive class) of the virtual screening models. This step certainly improves the computational efficiency of the various model validations. Furthermore, the distribution of active and inactive compounds in the projected space was analyzed and the results suggest that, based on the physicochemical properties (2D) of both classes, there was no significant discrimination found between the two classes. However, inactive compounds slightly have a large number of heavy atoms, a larger topological surface area and a higher number of largest chains compare to the active compounds (see the scatter plots provided in the supplementary material, Figure S3, Table S1). In terms of hydrogen bonding capability, a greater number of acceptors were observed in the active group compared to the inactive group, which have a greater number of hydrogen bond donors. This indication is consistent with the common structural features of RNase H active site inhibitors as reported previously [15], [17].

### Pharmacophore models of RNase H Inhibitor

**Figure 4:**
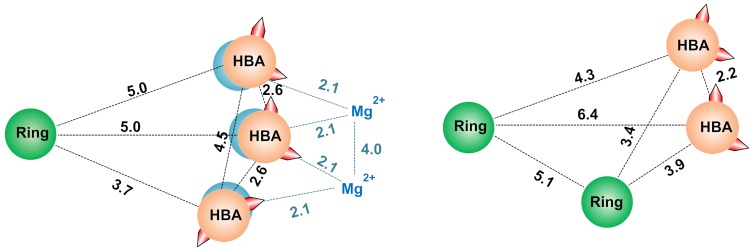
Pharmacophore hypothesis. Schematic representation of hypothesis 1 (left panel) and hypothesis 2 (right panel) for HIV-1 RT associated RNase H inhibitors. The metal ions chelation of N-hydroxyimides is shown in orange with distances measured by Klumpp et al.

For the development of a pharmacophore, we have considered 19 diverse inhibitors having pIC_50_ values from 5.0 to 8.6 against RNase H activity. Of the 19 compounds in the dataset, four were x-ray crystal bound inhibitors. The list of compounds used for the pharmacophore model is provided in the supplementary material (Figure S4). The Pharmacophore models were generated with two scenarios; in scenario 1, all 19 compounds were used for model building (default). In scenario 2, we further impose that the x-ray crystal bound ligands must match in the final hypothesis. The scenario 1 resulted in two hypotheses, which contains AAAR (*hypothesis-1*, survival score: 2.54, selectivity: 0.99 and matches: 11) and AARR (*hypothesis-2*, survival score: 2.40, selectivity: 1.12, matches: 11) features (A: Hydrogen bond acceptor, R: Ring). A schematic representation of the pharmacophore is shown in Figure 4. Scenario 2 also resulted in a AAAR feature, which is very similar to hypothesis 1 of scenario 1, except the inter-site distance. The inter-site distance measures the distance that separates the feature on the molecule from the centroid of the hypothesis feature. The hypotheses built from this study are in good agreement with previously established mechanism of RNase H inhibition, as most of the inhibitors chelates with magnesium ions in the active site. From the hypothesis it is observed that the distance between HBA1 and HBA3 was 4.5 Å and distance between HBA2 with HBA1 and HBA2 is 2.6 Å. This observation is in good agreement with previously reported distances of N-hydroxyimides chelated with metals, three of the two oxygen atoms in N-hydroxyimides is positioned with divalent metal ions at a distance of 4 Å (i.e., the distance between two metal ions) and each metal ions interact with oxygen atoms distance 2 Å) [22] (Figure 4). Similar results were reported for hydroxytropolones chelates with Mg^2+^ ions and the observed distance between two Mg^2+^ found to be 3.7 Å [59]. Although the compounds used for model building have diverse chemical scaffolds, the resulting hypothesis still had similar distance pattern that favors the metal chelation in the active site.

### Enrichment estimation from docking experiments

**Figure 5:**
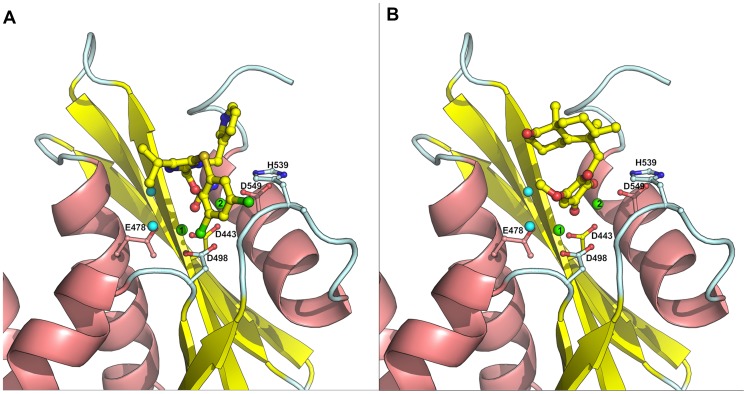
Inhibitors binding mode. Panel A. Capravirine, Panel B. Illimaquinone. RNase H active site is shown with important residues, water (cyan spheres) and magnesium ions (green spheres).

Reproducing the crystallographically observed conformation of the ligand is a minimum requirement to determine whether a docking setup is applicable to a given system. A receptor model obtained from Swiss-model was used for all docking experiments. Initially the N-hydroxy quinazolinedinone (NHQD) was prepared as described in the ligand preparation section and docked using the standard precision mode (SP) into the active site. Subsequently we compared the conformation and position with the bound ligand conformation measured in terms of the root-mean-square-deviation (RMSD). The measured RMSD of the docking pose was 0.23 Å and we observed similar interaction with the active site residues as the bound conformation (Figure 1B); for instance, HIS539 and Mg^2+^(2) interact with one of the three oxygen atoms of NHQD and the neighboring two oxygen atoms interact with the other Mg^2+^(1) ion and bound water molecules. It has previously been reported that the bound water molecules at the active site of RNase H plays a crucial role in RNA:DNA duplex digestion. It initiates the hydrolysis process through deproronation of water by Mg^2+^(1) and subsequently a OH^−^ ion attacks the phosphate of the RNA strand and leads to breaking of the phosphodiester bond [16], [17]. Subsequently, both sets of known active compounds, namely “subset_active_lit” and “subset_actives” were docked in the same manner and analyzed for its hydrogen-bonding (HB) network with residues, including water and metal. The results indicate that more than 56% of the inhibitors from both sets form a HB network with H539 and 40% of the inhibitors interact and form a HB network with two water molecules namely H_2_O_17 and H_2_O_24 in the active site. Compounds such as N-hydroxyl quinazolinedinone, naphthyridine, pryrimidinol analogues, capravirine (pIC50 = 8.07), PD126338 and Illimaquinone (pIC50 = 5.26) were found to strongly interact with magnesium ions in the RNase H active site (Figure 5).

As mentioned in the method section, in the present study the docking experiments were used to build virtual screening models, which will help in the identification of new compounds for RNase H inhibition. For validation purpose, the compounds in the set “subset_inactive” were also docked and scored. In this context, the docking scores (GlideScore) of both sets of active compounds were added into “subset_inactive”, and the enrichment factor (Eq. 5) was calculated using an in-build script in the Maestro module of the Schrödinger package. The enrichment factor is calculated using the number of known actives screened from inactive compounds at different % of database screening for various docking scenarios. A summary of the enrichment estimation is provided in Table 1. The receiver operator characteristic (ROC) was used to measure the effectiveness of the screening. From the enrichment analysis (Table 1), it is clear that all the docking scenarios (1–12) perform remarkably well with respect to finding actives from compounds that do not bind in the active site. In general, the enrichment effectiveness of the docking scenario score from SP (Standard Precision) performs reasonably well (ROC>0.80–0.91) compared to extra precision or high-throughput docking modes. We note that it is not completely appropriate to directly compare different docking modes which have been developed for different screening situation in drug design purpose e.g. the extra precision (XP) mode is computationally expensive (∼4 days for 1000 molecules using 4 CPU) and represents a significantly strict scoring procedure compared to SP. Models obtained from models 4–6 and 10–12 performed slightly better than models 1–3 and 7–9. Some models showed early and late enrichment, for instance, model 8 from “subset_actives_lit”, which found 53% of actives at 2% of screening (early enrichment) and 100% of actives at 77% screen. On the other hand, model 2 from subset_active found 30% of actives at 2% of screening and 100% of actives at 58% of screen (late enrichment). In addition, both active datasets were merged and the EF was calculated. The obtained merged models (13–18) were found to perform very similar to models 1–12 (see Table 1). From the enrichment analysis, one may chose a scenario that finds a considerable number of actives at early screening from large database.

### Machine learning classification models

Recently, machine learning (ML) methods have frequently begun to be used as virtual screening method, because these methods are able to relatively easy handle large datasets for building models to be applied to new predictions. In addition, ML methods also quickly find chemical patterns within the activity classes. Here, we developed various ML models for RNase H inhibition and non-inhibition using physicochemical properties (2D descriptors) and substructure fingerprints together with the BestFirst and the GeneticSearch algorithms as attribution selection methods. For classification, all active compounds (n = 741) and the compounds in “subset_inactives” were merged to build ML models. In total 18 models were developed to check which combination of descriptor and method performs better for classification (Table 2). The quality of the models was evaluated in terms of MCC and G-Mean of Test set prediction. Overall, models developed from random forest with descriptors and fingerprint performs better than other methods such as SVM or KNN. The models developed from other methods were efficient to predict non-inhibitors (>70%) compared to inhibitors (<70%). It is highly important to have models that are able to correctly predict both classes in a reasonably balanced manner, and not only correctly predicts one of the classes with high accuracy. The random forest model (4) using 111 descriptors predict 71% of inhibitors and 73% of non-inhibitors correctly with reasonably good coefficient (MCC = 0.44), and the quality is also reflected in the high G-mean score (0.72). In addition, if the random forest method in combination with the GeneticSearch algorithm on fingerprints (include MACCS and sub-structures) was used for model building (10), this resulted in a slightly reduced sensitivity and no change in the specificity. Overall the accuracy of the models (13–18) developed from combinations of fingerprint and descriptors did not change significantly (MCC = 0.43 and accuracy = 72%) compared to models developed only with descriptors (MCC = 0.44 and accuracy =  72%). However, the correct prediction [16] of inhibitors increased from 71 to 73% and non-inhibitor prediction slightly reduced from 73 to 71%. We achieved the best ML models with random forest in combination with 2D-descriptors.

**Table 2:** Summary of machine learning models from 2D descriptors and fingerprints (Test set prediction).

Descriptors	Attribution selection	MLMethods	Models	Confusion Matrix				Sens.	Spec.	G-M	MCC	OverallAccuracy
				TP	FN	TN	FP					
2D Descriptors	BestFirst(n = 37)	RF	1	141	87	218	74	0.62	0.75	0.68	0.37	0.69
		SVM	2	135	93	229	63	0.59	0.78	0.68	0.38	0.70
		KNN	3	140	88	210	82	0.61	0.72	0.66	0.33	0.67
	GenticSearch(n = 111)	RF	4	162	66	213	79	0.71	0.73	0.72	0.44	0.72
		SVM	5	143	85	228	64	0.63	0.78	0.70	0.41	0.71
		KNN	6	152	76	205	87	0.67	0.70	0.68	0.37	0.69
Fingerprints	BestFirst(n = 17)	RF	7	141	87	201	91	0.62	0.69	0.65	0.31	0.66
		SVM	8	108	120	238	54	0.47	0.82	0.62	0.31	0.67
		KNN	9	150	78	199	93	0.66	0.68	0.67	0.34	0.67
	GenticSearch(n = 55)	RF	10	154	74	212	80	0.68	0.73	0.70	0.40	0.70
		SVM	11	117	111	227	65	0.51	0.78	0.63	0.30	0.66
		KNN	12	146	82	196	96	0.64	0.67	0.66	0.31	0.66
Descriptors and Fingerprints	BestFirst(n = 51)	RF	13	160	68	198	94	0.70	0.68	0.69	0.38	0.69
		SVM	14	137	91	216	76	0.60	0.74	0.67	0.34	0.68
		KNN	15	139	89	215	77	0.61	0.74	0.67	0.35	0.68
	GenticSearch(n = 345)	RF	16	166	62	206	86	0.73	0.71	0.72	0.43	0.72
		SVM	17	149	79	219	73	0.65	0.75	0.70	0.40	0.71
		KNN	18	147	81	206	86	0.64	0.71	0.67	0.35	0.68
Han et al.	Fingerprints	DT^#^		640	130	98463	535	0.83	0.99	0.91	0.67	0.99
		DT^¢^		441	329	97923	1075	0.57	0.99	0.75	0.40	0.99

Note: RF: Random Forest, SVM: Support vector machine, KNN: Kappa nearest neighbor, TP: True Positive, FN: False negative, TN: True negative, FP: False positive, Sens.: Sensitivity, Spec.: Specificity, G-M: G-Mean, AUC: Area under curve, MCC: Mathews correlation coefficient, DT^#^: Decision tree training model and DT^¢^ : Decision tree 10-fold cross validation.

Han et al. [25] reported classification models using a decision tree method with approximately 99768 compounds, in which 770 compounds were inhibitors and 98998 compounds were non-inhibitors. Their training set showed an accuracy of 99% with significantly good sensitivity (83%) and specificity (99%). However, a ten-fold cross validation correctly predicts only 57% of the inhibitors, which is nearly the random prediction. This poor prediction might be due to an imbalanced distribution of active and inactive compounds in the dataset as this is a very common issue in QSAR/classification modeling with HTS dataset [60].

### Validation of queries from similarity search

**Figure 6:**
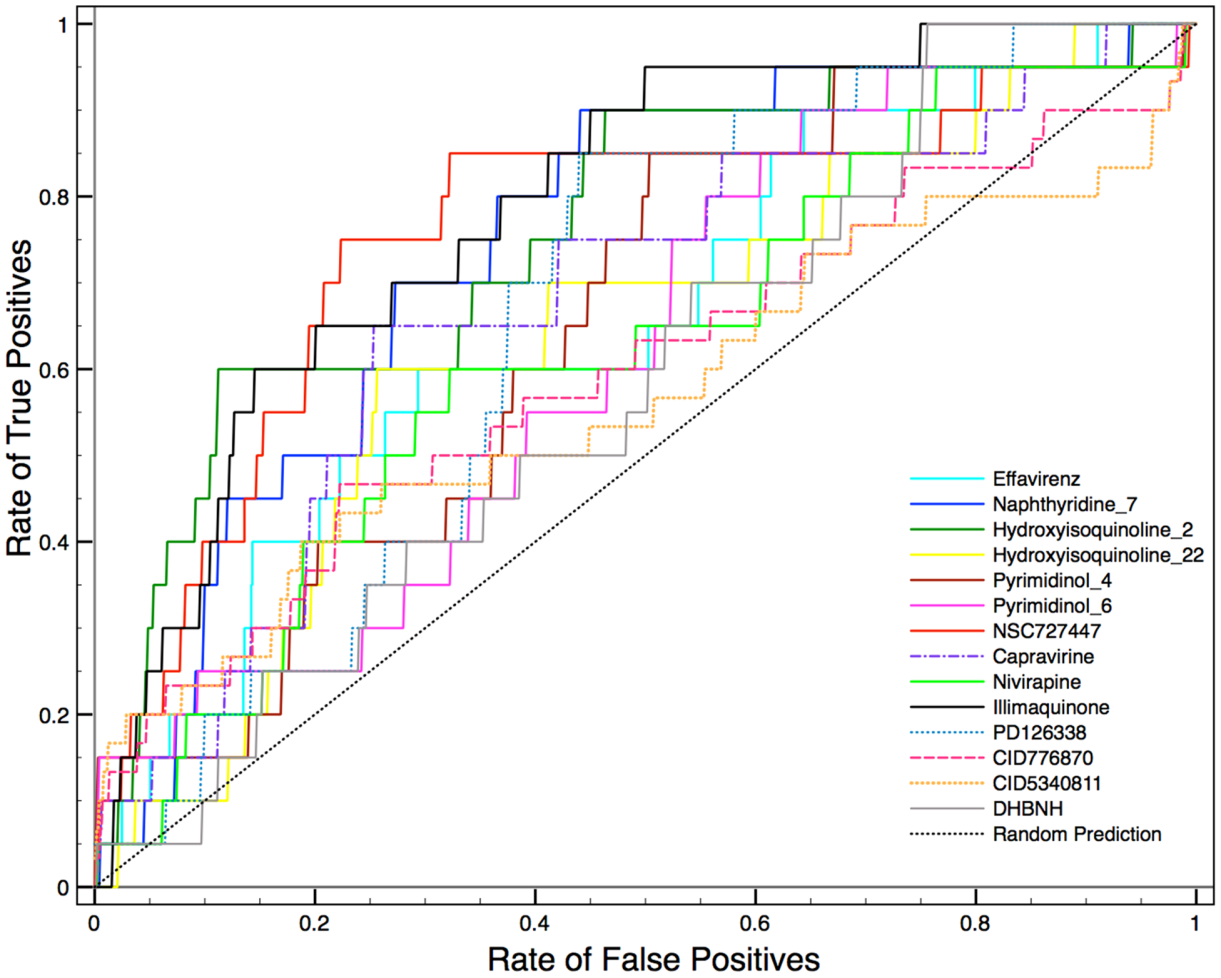
ROCS. Receiver operating characteristic curve comparison against selected query molecules used for structral similarity.

**Figure 7:**
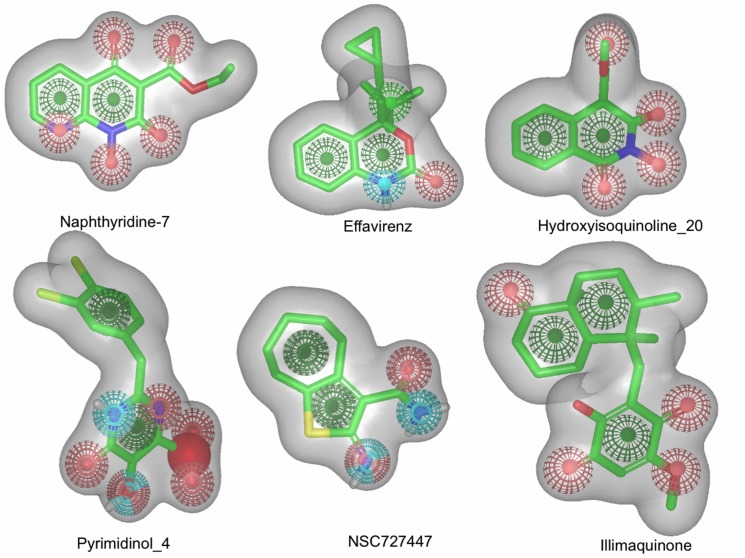
Shape and pharmacophore. The best query molecules from ROC validation are shown with hydrogen bonding donor (cyan), hydrogen-bonding acceptors (red), rings (green) and negatively charged (blue) features.

The rapid overlay of chemical structure (ROCS) tool is frequently being used for similarity based structure search in lead finding/optimization and it is relevant to validate which query molecule captures the majority of inhibitors shape and pharmacophoric information which discriminates from non-inhibitors. To the best of our knowledge, this is the first time that a large set of compounds has been used as query molecule for validation. The best query molecules could be used for virtual screening to identify chemically diverse RNase H inhibitors. To do this, we used the “subset_actives_lit” and “subset_active” datasets as query molecules and validated against each dataset using “subset_inactive” as decay dataset. For example, each one of the “subset_actives_lit” dataset compounds was used as a query molecule and checked if a particular inhibitor's shape and chemical pattern (ColorCombo) are able to find “subset_actives” (n = 20) from inactive compounds (n = 971). The enrichment factor and ROC were calculated (Table 3, Figure 6). It is clear from Table 3 that the quality of validation of each query molecule show a mixed performance, for example, some query molecules such as Nevirapine, DHBNH, PD126338, Pyrimidinol 6, CID776870 and CID 5340811 have good early enrichment, but their overall enrichment in screening is found to be poor, which is reflected from the low ROC. Compounds such as Efavirenz, Naphthyridine-7, Hydroisoquninoline-2/20, Pyrimidinol 4, NSC727447 and Capravirine showed good enrichment throughout the screening process and the ROC for these query molecules were significantly better (0.65–0.76). Interestingly, Illimaquinone shows poor performance in the beginning, but after 2% of the database screening, it seems to be very good, which is reflected in the good ROC value (0.8). Comparison of the best query molecules in terms of their shape and pharmacophoric features is shown in Figure 7. Looking at their color features, many of them have good agreement with hypothesis 1 (AAAR) derived from the pharmacophore modeling. Very recently, a compound DHBNH has been used as a query molecule for screening of the NCI database [26] and successfully found 4 micro molar inhibitors (2–22 µM). In the present similarity validation, DHBNH has also been used as query molecule, and shown good early enrichment. However the overall performance is nearly random (ROC = 0.5). From the results discussed from the ROCS validation, one can use the ROCS method not only to find similar compounds, but after thorough validation with a diverse set molecules, the query can potentially be used to find diverse molecules that will show same bioactivity.

**Table 3:** Validation of active subsets as a reference molecule for similarity search.

No	Query Compound	Enrichment Factor			ROC	No	Query Compound	Enrichment Factor			ROC
		0.5%	1%	2%				0.5%	1%	2%	
1	Nevirapine	6.63	4.98	2.51	0.63	26	Naphthyridine-2	0.00	0.06	2.24	0.70
2	Etraviridine	0.00	0.00	0.12	0.68	27	Naphthyridine-3	0.00	0.00	0.00	0.65
3	Naphthyridine-8	0.00	0.00	0.00	0.54	28	Naphthyridine-4	0.00	0.00	0.00	0.63
4	PD126338	10.27	5.13	2.57	0.65	29	Naphthyridine-6	0.00	0.00	0.00	0.63
5	Illimaquinone	0.03	0.73	5.02	0.80	30	Pyrimidinol-8	0.52	2.78	2.39	0.57
6	DHBNH	9.57	5.06	2.53	0.55	31	CID776870	13.86	11.62	6.79	0.60
7	BHMP07	0.00	0.00	0.00	0.41	32	CID 886534	0.00	0.00	0.00	0.55
8	Triazole-2d	0.00	0.00	0.00	0.60	33	CID 867945	0.00	0.00	0.00	0.42
9	Triazole-2e	0.00	0.00	0.14	0.62	34	CID 5348294	0.00	0.00	0.00	0.39
10	Efavirenz	10.27	5.13	3.21	0.66	35	CID 2258538	0.00	0.00	0.00	0.43
11	Naphthyridine-7	4.40	4.85	2.50	0.74	36	CID 5349528	0.00	0.00	0.00	0.53
12	Triazole	0.67	0.00	0.42	0.51	37	CID 5349588	0.00	0.00	0.00	0.47
13	Hydroxyisoquinoline-2	9.68	4.93	3.60	0.77	38	CID 5348178	2.80	3.14	1.64	0.47
14	Hydroxyisoquinoline-20	10.10	5.05	2.62	0.71	39	CID 2251798	0.00	0.00	0.00	0.51
15	Hydroxyisoquinoline-22	0.01	0.19	1.59	0.63	40	CID 5340811	23.34	15.98	8.63	0.58
16	Pyrimidinol4	30.03	15.08	7.54	0.66	41	CID 2263390	0.00	0.00	0.00	0.45
17	Pyrimidinol5	20.22	10.11	5.11	0.66	42	CID 2891629	0.00	0.00	0.00	0.45
18	Pyrimidinol6	28.28	14.77	7.39	0.62	43	CID 2257955	0.00	0.00	0.00	0.45
19	NSC727447	17.21	10.02	5.55	0.76	44	CID 767151	0.00	0.00	0.06	0.48
20	Triazole-2a	0.00	0.39	4.85	0.67	45	CID 1926286	0.00	0.00	0.00	0.48
21	Triazole-2b	0.00	0.00	0.00	0.65	46	CID 9586237	0.00	0.00	0.00	0.37
22	Nitrofuron-10	0.00	0.00	0.00	0.52	47	CID 95786	0.00	0.00	0.07	0.44
23	Merk Cpd	0.00	0.00	0.01	0.68	48	CID 1730618	0.00	0.00	0.00	0.56
24	Capravirine	11.43	10.13	5.17	0.68	49	CID 1732694	0.00	0.00	0.00	0.52
25	GW8248	0.00	0.00	0.00	0.52	50	CID 2263150	0.00	0.00	0.00	0.48

### Fingerprints for ligands and proteins (FLAP) models

**Figure 8:**
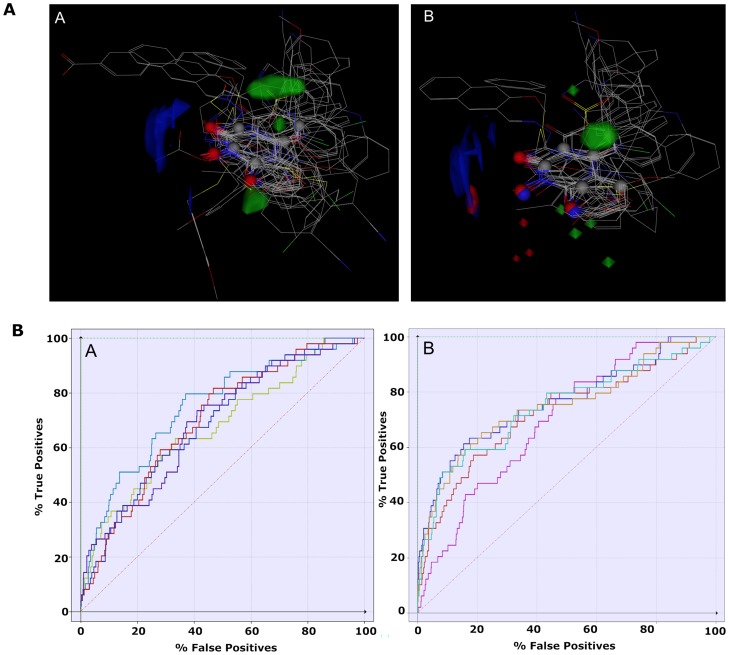
FLAP Models. Panel A. Pseudomolecule (Pharmacophoric atoms) shown (left) in gray/blue/red spheres with aligned molecules (wire representation), modified model 1 (right). GRID 3D molecule interaction fields (MIFs) of models are shown as follows: The green contours correspond to the DRY probe (energy level −0.63 kcal/mol), blue contours correspond to the N1 probe (energy level −3.83 kcal/mol) and red contours correspond to the O probe (energy level −1.50 kcal/mol). Panel B. Enrichment plot of screening for 20 known actives from 971 inactive compounds from the PubChem database. Enrichment plot of model 1–4 (left), Enrichment plot of modified model 1–4 (right).

The FLAP program was used for molecular interaction fields (MIFs)-based pharmacophore virtual screening. Here the subset_active_lit dataset was used to develop pharmacophore and subsequently applied for validation. FLAP generates possible ensembles of conformations for each active molecule and keeps the pharmacophorically similar conformers. Subsequently, the conformers were aligned based on the pruned tree search. The aligned models were used to generate a *pharmacophorically psuedomolecule*, which consist of pharmacophoric points. The quality of an aligned model was assessed using the fitness score and AUC value. The best 5 aligned models were analyzed. Each model uses different sets of molecules for alignment and the fitness score ranges were from 0.85 to 0.95 (Table 4, Figure 8). The best enrichment was found from model 3 using the Glob-Prod score with an AUC of 0.73 and followed by models 1, 4, 5 which are based on Glob-Prod and H (denotes shape) similarity parameters with an AUC of 0.69. However, the quality of all models was improved significantly when removing compounds that have less global similarity score (also called the S-score) in every model, for instance, model 1 was refined by removing 11 actives (having S-score less than 0.8) from the alignment. The resulting pharmacophore model shows relatively good enrichment using Glob-Prod parameters and yielding an AUC of 0.76. In a similar manner, all the remaining models were refined and showed to possess good enrichment using various similarity parameters such as N1 (hydrogen bond acceptors), O (hydrogen bond donor) and H (shape) score.

**Table 4:** Comparison of various FLAP-pharmacophore models.

Model	Alignment fitness	Screening				
		Contributing Parameters	AUC	Compounds in edited Model	Contributing Parameters	AUC
1	0.95	Glob-Prod	0.69	19	Glob-Prod	0.76
2	0.94	H+N1+N	0.67	20	H	0.69
3	0.92	Glob-Prod	0.73	18	Glob-Sum	0.72
4	0.89	H	0.69	15	H+O+N1	0.75
5	0.85	H	0.69	15	H+N1+H	0.74

Note: H: Shape, O: Hydrogen bond acceptor, N1: Hydrogen bond donor, DRY: Hydrophobic, Glob-Prod: Global Product scores, Glob_Sum: Global Sum score, AUC: Area under curve.

### Comparison of the developed models

**Figure 9:**
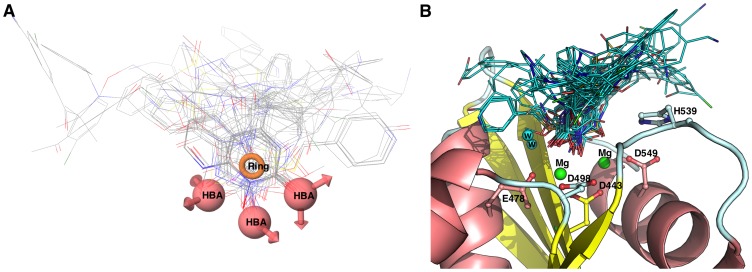
Comparison of the pharmacophore model with docking experiment. Panel A. First ranked docking poses superimposed with the pharmacophore (Hypothesis 1, AAAR). Panel B. The first ranked docking pose is shown in the active site of RNase H and important residues are highlighted, including magnesium ions (green sphere) and water (cyan sphere).

Comparison of the efficacy and efficiency of the developed models needs to be done with caution since the methods used in this study are conceptually different and possesses their own pros and cons. However, from a virtual screening point of view the models were compared in terms of how quickly they were able to find known inhibitors from a large set of non-inhibitors. Among the methods used to efficiently identify new RNase H inhibitors, the best performances were achieved with structure-based models such as docking (83–91%), followed by FLAP (76%), similarity search (74–77%), and machine learning e.g., random forest (72%). A summary of the time scale for new predictions based on the use of the different methods is provided in Table 5. The docking experiments produced the models (2, 5, 8, 11, 14, 17) with the highest performance using standard precision (SP). Considering the methods efficiency for new prediction, ligand based methods such as similarity search and machine learning models are very efficient, for instance setting up the machine learning based models for new prediction would not take more than an hour for approximately 20000 compounds (this process includes dataset set preprocess, descriptors calculation and new prediction). However, the efficacy of ML models is slightly less than the other methods. Compounds such as NSC727447, Hydroxyisoquninoline_2/20, Naphthyridine_7 and pyrimidinol_4/5 could be used as reference molecules for new inhibitor search. From the ROCS experiment, it was noted that these molecules potentially captures most of the available known inhibitor structural information (ROC = 66–77%) and observed very good early enrichment in the screening experiment. From an efficacy point of view in virtual screening, ROC and ML methods could be the methods of choice. Alternatively, docking experiment with standard precision setup (in Glide) could be used for high enrichment in the virtual screening process. In addition docking experiments not only take advantage over the other methods in terms of high enrichment, but also proposes energetically favorable poses of the ligands in the binding pocket of a protein and thereby predicts key interactions. However, from an efficiency point of view docking predictions might be slow for large chemical database searches. Although the FLAP model predicts reasonably well (ROC = 0.76), the efficiency could be slow as compared to the other models. It is important to analyze if the ligand based pharmacophore model and the best ranked docking pose of the validation compounds suggest similar biological interactions or not. In order to compare this, the first ranked docking pose (XP scoring) for all validation compounds were superimposed with hypothesis 1 (AAAR). The result suggests that the information derived from the ligand-based pharmacophore model (AAAR) merely reflects the complementary receptor as shown in Figure 9. As mentioned previously, hydrogen bonding acceptors play an essential role in binding with metal chelation.

**Table 5:** Approximate time scale for new predictions using the developed models.

Method	Model	Overall Accuracy	No. Molecules (per hour)	Efficacy vs. Efficiency
Pharmacophore	Hypothesis 1/2	AAAR	>10,000	Efficient
Docking	17	0.90	∼1000	Efficient
Docking	18	0.81	∼100	Efficacy
ROCS	Queries	∼0.70	>100,000	Efficient
FLAP	1	0.76	∼10,000	Efficacy
Machine Learning	4, 10	70–72%	>20,000	Efficient

Note: The approximate time scale includes the dataset processing (such as 3D generation, energy minimization, descriptor calculation) and applications for new predictions. Queries: molecules Naphthyridine_7, Effavirenz, Hydroxyisoquinoline_20, Pryimidinol_4, NSC 727447 and Ilimaquinone.

## Conclusions

In the present study we have developed virtual screening models from various conceptually different computational methods (structure and ligand based) to efficiently identify HIV-1 RT associated RNase Inhibitors. Models based on the docking experiments are superior to other ligand-based models. Considering efficacy and efficiency, models trained using random forest with combination of 2D-descriptors and structural fingerprints are highly efficient methods, however, docking experiments correctly predicts more than 80% and are relatively less time consuming, and these could thus be useful for new prediction. In addition protein-ligand key interaction information from docking could be useful for lead optimization e.g. after virtual screening.

Moreover, the hypothesis generated from pharmacophore modeling and molecular interaction fields from the FLAP experiment illustrates the importance of hydrogen bonding acceptors and hydrophobic features. These features are in good agreement with the previously reported x-ray structures of HIV-1 RT associated RNase H. The pharmacophore model suggests that the inter-distance between hydrogen bond acceptors play a key role in inhibition of RNase H domain through metal chelation. The observed distances between HB acceptors are consistent with previous experimentally measured distances. As discussed, the docking procedure remains the method of choice for virtual screening; however, random forest and similarity search method could also be used alternatively to efficiently screen large database in order to establish new RNase H inhibitors for highly active retroviral therapy.

## Supporting Information

Figure S1 **List of compounds used as known actives.**
(TIFF)

Figure S2 **Representative compounds for each clusters.**
(TIFF)

Figure S3 **Scatter plots of actives and inactive compounds based on the physicochemical properties.**
(TIFF)

Figure S4 **List of compounds used for pharmacophore model building.**
(TIFF)

Table S1 **Molecular Properties used for comparison of active and inactive.**
(DOCX)

File S1 **Structure and activity for the subsets used in this study are provided separately as “Subset.sdf”.**
(ZIP)

## References

[1] UNAIDS (2012) Global report: UNAIDS report on the global AIDS epidemic 2012, Joint United Nations Programme on HIV/AIDS.

[2] Fauci AS (2003) HIV and AIDS: 20 years of science. Nat Med 9: 839–843. 12835701 10.1038/nm0703-839

[3] De Clercq E (2002) New developments in anti-HIV chemotherapy. Biochimica Et Biophysica Acta-Molecular Basis of Disease 1587: 258–275. 10.1016/s0925-4439(02)00089-3 12084468

[4] Zdanowicz MM (2006) The pharmacology of HIV drug resistance. Am J Pharm Educ 70: 100. 17149429 10.5688/aj7005100 PMC1637011

[5] Darbyshire J (1995) Perspectives in drug therapy of HIV infection. Drugs 49 Suppl 1: 1–3 discussion 38-40. 10.2165/00003495-199500491-00003 7614897

[6] Bean P (2005) New drug targets for HIV. Clin Infect Dis 41 Suppl 1: S96–100. 16265623 10.1086/429504

[7] Imamichi T (2004) Action of anti-HIV drugs and resistance: reverse transcriptase inhibitors and protease inhibitors. Curr Pharm Des 10: 4039–4053. 15579086 10.2174/1381612043382440

[8] Hartman TL, Buckheit RW Jr (2012) The Continuing Evolution of HIV-1 Therapy: Identification and Development of Novel Antiretroviral Agents Targeting Viral and Cellular Targets. Mol Biol Int 2012: 401965. 22848825 10.1155/2012/401965 PMC3400388

[9] Greene WC, Debyser Z, Ikeda Y, Freed EO, Stephens E, et al (2008) Novel targets for HIV therapy. Antiviral Res 80: 251–265. 18789977 10.1016/j.antiviral.2008.08.003

[10] Martinez LJ (2002) The need for novel targets and approaches to HIV therapy. Res Initiat Treat Action 8: 23–25. 12489523

[11] Hang JQ, Li Y, Yang Y, Cammack N, Mirzadegan T, et al (2007) Substrate-dependent inhibition or stimulation of HIV RNase H activity by non-nucleoside reverse transcriptase inhibitors (NNRTIs). Biochem Biophys Res Commun 352: 341–350. 17113568 10.1016/j.bbrc.2006.11.018

[12] Julias JG, McWilliams MJ, Sarafianos SG, Alvord WG, Arnold E, et al (2003) Mutation of amino acids in the connection domain of human immunodeficiency virus type 1 reverse transcriptase that contact the template-primer affects RNase H activity. J Virol 77: 8548–8554. 12857924 10.1128/JVI.77.15.8548-8554.2003 PMC165255

[13] Sarafianos SG, Das K, Hughes SH, Arnold E (2004) Taking aim at a moving target: designing drugs to inhibit drug-resistant HIV-1 reverse transcriptases. Curr Opin Struct Biol 14: 716–730. 15582396 10.1016/j.sbi.2004.10.013

[14] Himmel DM, Maegley KA, Pauly TA, Bauman JD, Das K, et al (2009) Structure of HIV-1 reverse transcriptase with the inhibitor beta-Thujaplicinol bound at the RNase H active site. Structure 17: 1625–1635. 20004166 10.1016/j.str.2009.09.016 PMC3365588

[15] Lansdon EB, Liu Q, Leavitt SA, Balakrishnan M, Perry JK, et al (2011) Structural and binding analysis of pyrimidinol carboxylic acid and N-hydroxy quinazolinedione HIV-1 RNase H inhibitors. Antimicrob Agents Chemother 55: 2905–2915. 21464257 10.1128/AAC.01594-10 PMC3101433

[16] Davies JF 2nd, Hostomska Z, Hostomsky Z, Jordan SR, Matthews DA (1991) Crystal structure of the ribonuclease H domain of HIV-1 reverse transcriptase. Science 252: 88–95. 1707186 10.1126/science.1707186

[17] Ilina T, Labarge K, Sarafianos SG, Ishima R, Parniak MA (2012) Inhibitors of HIV-1 Reverse Transcriptase-Associated Ribonuclease H Activity. Biology (Basel) 1: 521–541. 23599900 10.3390/biology1030521 PMC3627382

[18] Klumpp K, Mirzadegan T (2006) Recent progress in the design of small molecule inhibitors of HIV RNase H. Curr Pharm Des 12: 1909–1922. 16724956 10.2174/138161206776873653

[19] Tramontano E, Di Santo R (2010) HIV-1 RT-associated RNase H function inhibitors: Recent advances in drug development. Current Medicinal Chemistry 17: 2837–2853. 20858167 10.2174/092986710792065045

[20] Felts AK, Labarge K, Bauman JD, Patel DV, Himmel DM, et al (2011) Identification of alternative binding sites for inhibitors of HIV-1 ribonuclease H through comparative analysis of virtual enrichment studies. J Chem Inf Model 51: 1986–1998. 21714567 10.1021/ci200194w PMC3159817

[21] Budihas SR, Gorshkova I, Gaidamakov S, Wamiru A, Bona MK, et al (2005) Selective inhibition of HIV-1 reverse transcriptase-associated ribonuclease H activity by hydroxylated tropolones. Nucleic Acids Res 33: 1249–1256. 15741178 10.1093/nar/gki268 PMC552956

[22] Klumpp K, Hang JQ, Rajendran S, Yang Y, Derosier A, et al (2003) Two-metal ion mechanism of RNA cleavage by HIV RNase H and mechanism-based design of selective HIV RNase H inhibitors. Nucleic Acids Res 31: 6852–6859. 14627818 10.1093/nar/gkg881 PMC290251

[23] Shaw-Reid CA, Munshi V, Graham P, Wolfe A, Witmer M, et al (2003) Inhibition of HIV-1 ribonuclease H by a novel diketo acid, 4-[5-(benzoylamino)thien-2-yl]-2,4-dioxobutanoic acid. J Biol Chem 278: 2777–2780. 12480948 10.1074/jbc.C200621200

[24] Pubchem BioAssay Database. Available: http://www.pubchem.ncbi.nlm.nih.gov. Accessed 2013 Mar 20.

[25] Han L, Wang Y, Bryant SH (2008) Developing and validating predictive decision tree models from mining chemical structural fingerprints and high-throughput screening data in PubChem. BMC Bioinformatics 9: 401. 18817552 10.1186/1471-2105-9-401 PMC2572623

[26] Distinto S, Esposito F, Kirchmair J, Cardia MC, Gaspari M, et al (2012) Identification of HIV-1 reverse transcriptase dual inhibitors by a combined shape-, 2D-fingerprint- and pharmacophore-based virtual screening approach. Eur J Med Chem 50: 216–229. 22361685 10.1016/j.ejmech.2012.01.056

[27] Vasanthanathan P, Lastdrager J, Oostenbrink C, Commandeur JNM, Vermeulen NPE, et al (2011) Identification of CYP1A2 ligands by structure-based and ligand-based virtual screening. MedChemComm 2: 853–859.

[28] Schneider G, Bohm HJ (2002) Virtual screening and fast automated docking methods. Drug Discovery Today 7: 64–70. 11790605 10.1016/s1359-6446(01)02091-8

[29] McInnes C (2007) Virtual screening strategies in drug discovery. Curr Opin Chem Biol 11: 494–502. 17936059 10.1016/j.cbpa.2007.08.033

[30] Billamboz M, Bailly F, Barreca ML, De Luca L, Mouscadet JF, et al (2008) Design, synthesis, and biological evaluation of a series of 2-hydroxyisoquinoline-1,3(2H,4H)-diones as dual inhibitors of human immunodeficiency virus type 1 integrase and the reverse transcriptase RNase H domain. J Med Chem 51: 7717–7730. 19053754 10.1021/jm8007085

[31] Billamboz M, Bailly F, Lion C, Touati N, Vezin H, et al (2011) Magnesium chelating 2-hydroxyisoquinoline-1,3(2H,4H)-diones, as inhibitors of HIV-1 integrase and/or the HIV-1 reverse transcriptase ribonuclease H domain: discovery of a novel selective inhibitor of the ribonuclease H function. J Med Chem 54: 1812–1824. 21366258 10.1021/jm1014692

[32] Borkow G, Fletcher RS, Barnard J, Arion D, Motakis D, et al (1997) Inhibition of the ribonuclease H and DNA polymerase activities of HIV-1 reverse transcriptase by N-(4-tert-butylbenzoyl)-2-hydroxy-1-naphthaldehyde hydrazone. Biochemistry 36: 3179–3185. 9115994 10.1021/bi9624696

[33] Di Grandi M, Olson M, Prashad AS, Bebernitz G, Luckay A, et al (2010) Small molecule inhibitors of HIV RT Ribonuclease H. Bioorg Med Chem Lett 20: 398–402. 19939680 10.1016/j.bmcl.2009.10.043

[34] Hazuda DJ, Anthony NJ, Gomez RP, Jolly SM, Wai JS, et al (2004) A naphthyridine carboxamide provides evidence for discordant resistance between mechanistically identical inhibitors of HIV-1 integrase. Proc Natl Acad Sci U S A 101: 11233–11238. 15277684 10.1073/pnas.0402357101 PMC509174

[35] Kirschberg TA, Balakrishnan M, Squires NH, Barnes T, Brendza KM, et al (2009) RNase H active site inhibitors of human immunodeficiency virus type 1 reverse transcriptase: design, biochemical activity, and structural information. J Med Chem 52: 5781–5784. 19791799 10.1021/jm900597q

[36] Su HP, Yan Y, Prasad GS, Smith RF, Daniels CL, et al (2010) Structural basis for the inhibition of RNase H activity of HIV-1 reverse transcriptase by RNase H active site-directed inhibitors. J Virol 84: 7625–7633. 20484498 10.1128/JVI.00353-10 PMC2897604

[37] Tramontano E, Esposito F, Badas R, Di Santo R, Costi R, et al (2005) 6-[1-(4-Fluorophenyl)methyl-1H-pyrrol-2-yl)]-2,4-dioxo-5-hexenoic acid ethyl ester a novel diketo acid derivative which selectively inhibits the HIV-1 viral replication in cell culture and the ribonuclease H activity in vitro. Antiviral Res 65: 117–124. 15708638 10.1016/j.antiviral.2004.11.002

[38] Wendeler M, Lee HF, Bermingham A, Miller JT, Chertov O, et al (2008) Vinylogous ureas as a novel class of inhibitors of reverse transcriptase-associated ribonuclease H activity. ACS Chem Biol 3: 635–644. 18831589 10.1021/cb8001039 PMC2941776

[39] Williams PD, Staas DD, Venkatraman S, Loughran HM, Ruzek RD, et al (2010) Potent and selective HIV-1 ribonuclease H inhibitors based on a 1-hydroxy-1,8-naphthyridin-2(1H)-one scaffold. Bioorg Med Chem Lett 20: 6754–6757. 20869872 10.1016/j.bmcl.2010.08.135

[40] Yanagita H, Urano E, Matsumoto K, Ichikawa R, Takaesu Y, et al (2011) Structural and biochemical study on the inhibitory activity of derivatives of 5-nitro-furan-2-carboxylic acid for RNase H function of HIV-1 reverse transcriptase. Bioorg Med Chem 19: 816–825. 21193314 10.1016/j.bmc.2010.12.011

[41] Parniak MA, Min KL, Budihas SR, Le Grice SF, Beutler JA (2003) A fluorescence-based high-throughput screening assay for inhibitors of human immunodeficiency virus-1 reverse transcriptase-associated ribonuclease H activity. Anal Biochem 322: 33–39. 14705777 10.1016/j.ab.2003.06.001

[42] Standardizer (v5.12.2) (2013), ChemAxon. Available: http://www.chemaxon.com.

[43] Hawkins PC, Skillman AG, Warren GL, Ellingson BA, Stahl MT (2010) Conformer generation with OMEGA: algorithm and validation using high quality structures from the Protein Databank and Cambridge Structural Database. J Chem Inf Model 50: 572–584. 20235588 10.1021/ci100031x PMC2859685

[44] Michael RB, Nicolas C, Fabian D, Thomas RG, Tobias KO, et al.. (2009) KNIME-The Konstanz Information Miner ACM SIGKDD Explorations Newsletter. New York, USA: ACM pp. 31.

[45] Canvas (v1.5) (2012) Schrödinger, LLC, New York, NY.

[46] Witten IH, Frank E (2005) Data Mining: Practical machine learning tools and techniques. San Francisco: Morgan Kaufmann.

[47] Hall M, Frank E, Holmes H, Pfahringer B, Reutemann P, et al (2009) The WEKA Data Mining Software: An Update. SIGKDD Explorations 11.

[48] Yap CW (2011) PaDEL-descriptor: an open source software to calculate molecular descriptors and fingerprints. J Comput Chem 32: 1466–1474. 21425294 10.1002/jcc.21707

[49] Dixon SL, Smondyrev AM, Knoll EH, Rao SN, Shaw DE, et al (2006) PHASE: a new engine for pharmacophore perception, 3D QSAR model development, and 3D database screening: 1. Methodology and preliminary results. J Comput Aided Mol Des 20: 647–671. 17124629 10.1007/s10822-006-9087-6

[50] Schrödinger Suite (2013) Schrödinger LLC., Portland, USA. Available: http://www.schrodinger.com.

[51] vROC OEChem, (v1.7.2) (2010), OpenEye Scientific Software, Inc., Santa Fe, NM, USA. Available: http://www.eyesopen.com.

[52] Grant JA, Gallardo MA, Pickup BT (1996) A fast method of molecular shape comparison: A simple application of a Gaussian description of molecular shape. J Comput Chem 17: 1653–1666.

[53] Baroni M, Cruciani G, Sciabola S, Perruccio F, Mason JS (2007) A common reference framework for analyzing/comparing proteins and ligands. Fingerprints for Ligands and Proteins (FLAP): theory and application. J Chem Inf Model 47: 279–294. 17381166 10.1021/ci600253e

[54] Arnold K, Bordoli L, Kopp J, Schwede T (2006) The SWISS-MODEL workspace: a web-based environment for protein structure homology modelling. Bioinformatics 22: 195–201. 16301204 10.1093/bioinformatics/bti770

[55] Madhavi Sastry G, Adzhigirey M, Day T, Annabhimoju R, Sherman W (2013) Protein and ligand preparation: parameters, protocols, and influence on virtual screening enrichments. J Comput Aided Mol Des 27: 221–234. 23579614 10.1007/s10822-013-9644-8

[56] Li H, Robertson AD, Jensen JH (2005) Very fast empirical prediction and rationalization of protein pKa values. Proteins 61: 704–721. 16231289 10.1002/prot.20660

[57] Friesner RA, Banks JL, Murphy RB, Halgren TA, Klicic JJ, et al (2004) Glide: a new approach for rapid, accurate docking and scoring. 1. Method and assessment of docking accuracy. J Med Chem 47: 1739–1749. 15027865 10.1021/jm0306430

[58] Shelley JC, Cholleti A, Frye LL, Greenwood JR, Timlin MR, et al (2007) Epik: a software program for pK(a) prediction and protonation state generation for drug-like molecules. J Comput Aided Mol Des 21: 681–691. 17899391 10.1007/s10822-007-9133-z

[59] Didierjean J, Isel C, Querre F, Mouscadet JF, Aubertin AM, et al (2005) Inhibition of human immunodeficiency virus type 1 reverse transcriptase, RNase H, and integrase activities by hydroxytropolones. Antimicrob Agents Chemother 49: 4884–4894. 16304149 10.1128/AAC.49.12.4884-4894.2005 PMC1315922

[60] Singh N, Chaudhury S, Liu R, AbdulHameed MD, Tawa G, et al (2012) QSAR classification model for antibacterial compounds and its use in virtual screening. J Chem Inf Model 52: 2559–2569. 23013546 10.1021/ci300336v

