# Supplementary figures and images for: Virtual Screening Models for Prediction of HIV-1 RT Associated RNase H Inhibition
Source: PLoS One. 2013 Sep 16;8(9):e73478. doi: 10.1371/journal.pone.0073478 (PMC3774690; doi:10.1371/journal.pone.0073478)

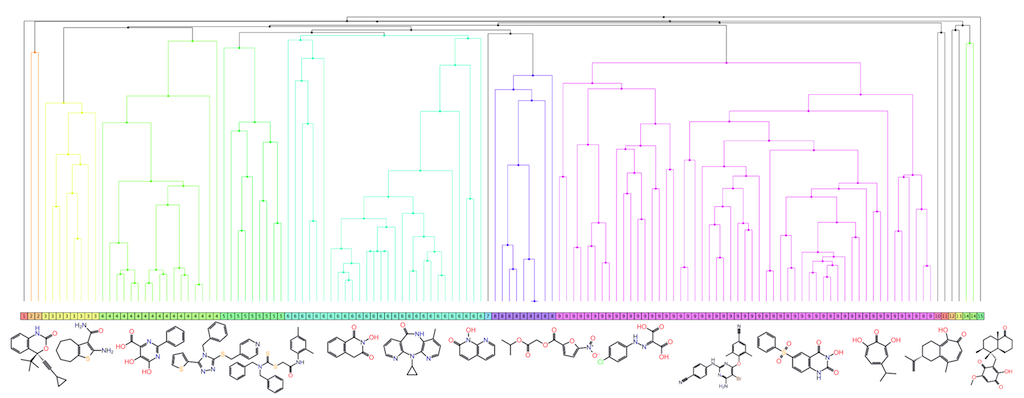

Supplement: Figure S2 — Representative compounds for each clusters. (TIFF) [file pone.0073478.s002.tif]

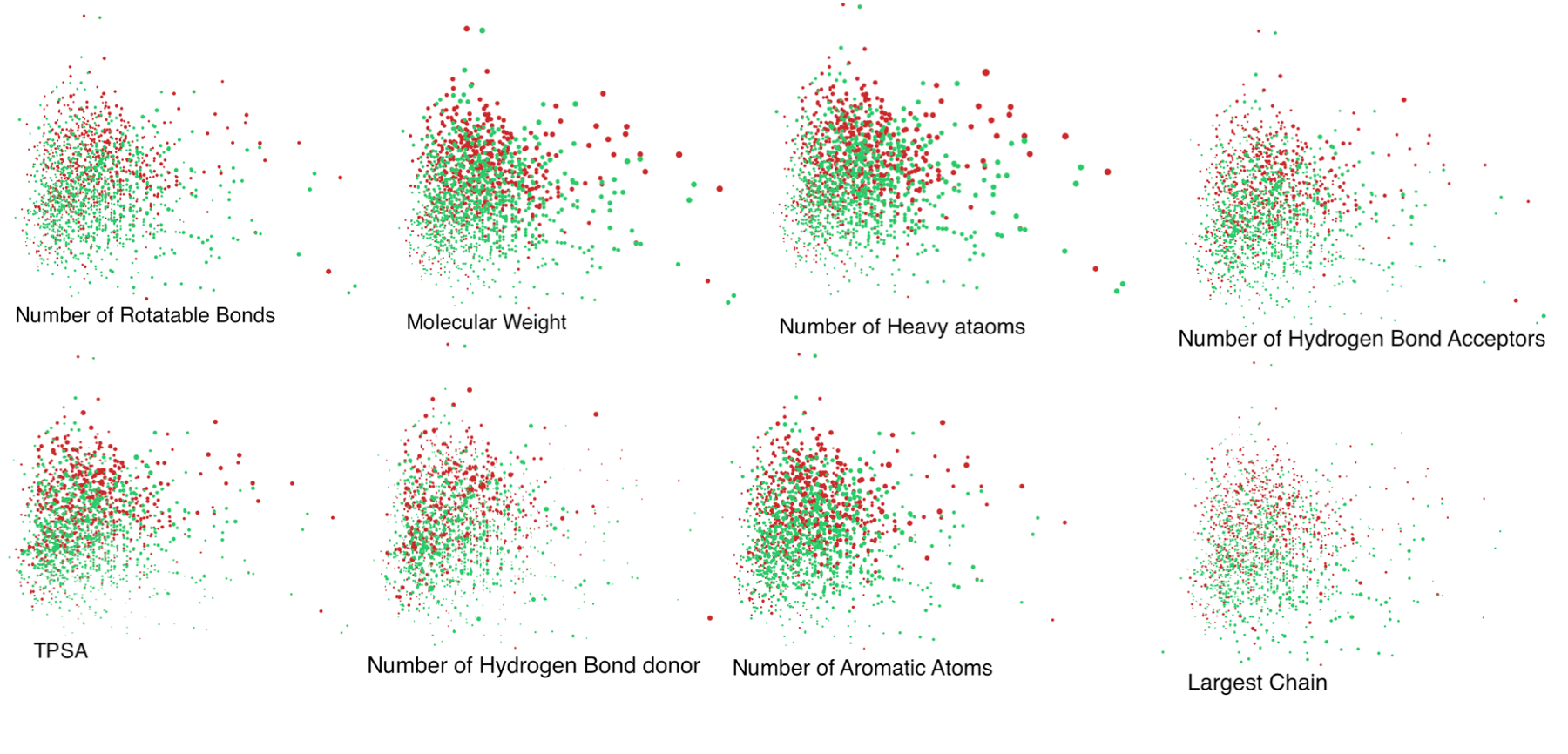

Supplement: Figure S3 — Scatter plots of actives and inactive compounds based on the physicochemical properties. (TIFF) [file pone.0073478.s003.tif]
