# Supplementary material for: Virtual Screening Models for Prediction of HIV-1 RT Associated RNase H Inhibition
Source: PLoS One. 2013 Sep 16;8(9):e73478. doi: 10.1371/journal.pone.0073478 (PMC3774690; doi:10.1371/journal.pone.0073478)
Supplement: Table S1 — Molecular Properties used for comparison of active and inactive. (DOCX) [file pone.0073478.s005.docx]

**Table S1**

| Property | Minimum (Small dot) | Maximum (large dot) |
| --- | --- | --- |
| Number of Aromatic atoms | 0 | 38 |
| Number of HB Donors | 0 | 6 |
| Number of HB Acceptors | 0 | 16 |
| Largest Chain | 0 | 39 |
| Number of Rotatable Bonds | 0 | 29 |
| TPSA | 6 | 265 |
| Molecular weight | 149 | 695 |
| Number of Heavy atoms | 10 | 48 |
